# Exercise Intervention for Bone Metastasis: Safety, Efficacy and Method of Delivery

**DOI:** 10.3390/cancers15061786

**Published:** 2023-03-15

**Authors:** Huong Duong, Meegan Walker, Michelle Maugham-Macan

**Affiliations:** School of Health and Behavioural Sciences, University of the Sunshine Coast, Sippy Downs, QLD 4556, Australia

**Keywords:** bone metastasis, exercise, safety, fracture risk, remote, in-person

## Abstract

**Simple Summary:**

This paper reviews the current literature on the safety of exercise for patients with bone metastases. It compares the effectiveness of in-person vs. remote delivery of exercise intervention and reports on adverse outcomes such as fractures in these studies.

**Abstract:**

Background: The benefits of exercise for patients with cancer are well-established, however, for patients with bone metastases, exercise as adjuvant therapy is underutilised due to concerns for safety, efficacy and other barriers such as the method of delivery. This scoping review explores these barriers by reviewing the results of clinical trials conducted on participants with bone metastases. Methods: A thorough literature search was undertaken using PubMed, Scopus, NIH Clinical Trials and Google Scholar databases. Articles that involved an exercise intervention and patients with bone metastases were included. Data were pooled, charted, analysed and reported according to PRISMA-ScR standards. Results: A total of 26 trials were reviewed with interventions that included aerobic and resistance training. Only three serious adverse events occurred, not likely related to bone metastases. Nine trials (34.6%) involved unsupervised exercise sessions. Remote exercise delivery had an average of 80.3% compliance, rivalling in-person and mixed supervision. The results of this review reaffirm that exercise helps improve functional capacity, muscle strength, lean mass and cardiovascular function, and is safe in patients with bone metastases irrespective of in-person or remote delivery. Conclusions: Exercise therapy, whether delivered in person or remotely, is safe and efficacious for patients with bone metastases.

## 1. Introduction

It is well known that exercise benefits cancer patients [1,2,3]. However, the evidence needed to extend this finding to patients with bone metastases has only been established in recent years. Previously, physicians have been hesitant to prescribe exercise for patients with bone metastases, due to a variety of concerns, of which potential skeletal complications are the primary deterrent. Less than half the professionals responsible for the treatment of patients with bone metastases were confident to recommend this type of adjuvant therapy [1]. Given the benefits of exercise for patients with bone metastases emerging in this rapidly expanding field of research, establishing safety and practicality of exercise in this population is of utmost importance.

With patient safety a concern, in-person delivery under the supervision of an accredited exercise physiologist is a responsible option. However, for some patients with bone metastases, attending regular, in-person sessions may not be feasible.

This scoping review examines the safety and efficacy of exercise intervention with a focus on remote delivery exercise therapy to benefit patients who, for various reasons, cannot access in-person therapy. 

## 2. Materials and Methods

### 2.1. Protocol

The methodological framework for this scoping review was based on procedures developed by Arksey and O’Malley and integrated additional scoping review recommendations made by Levac et al. [4,5]. The results were reported according to the PRISMA-ScR (Preferred Reporting Items for Systematic reviews and meta-analyses extension for Scoping Reviews) guidelines. 

There were five broad stages of this scoping review. 

Stage 1: Identify the research question. 

Our research question was “can remote-based exercise therapy be safely and efficaciously used in patients with bone metastases?” 

Stages 2 and 3: Identifying and selecting relevant studies. 

The database search was conducted by the authors. Study selection and review took approximately 4 weeks. The following electronic databases were searched: (1) PubMed, (2) Scopus, (3) NIH Clinical Trials and (4) Google Scholar. We limited our search to documents published in English between 2000 and 2022. 

**Figure 1:**
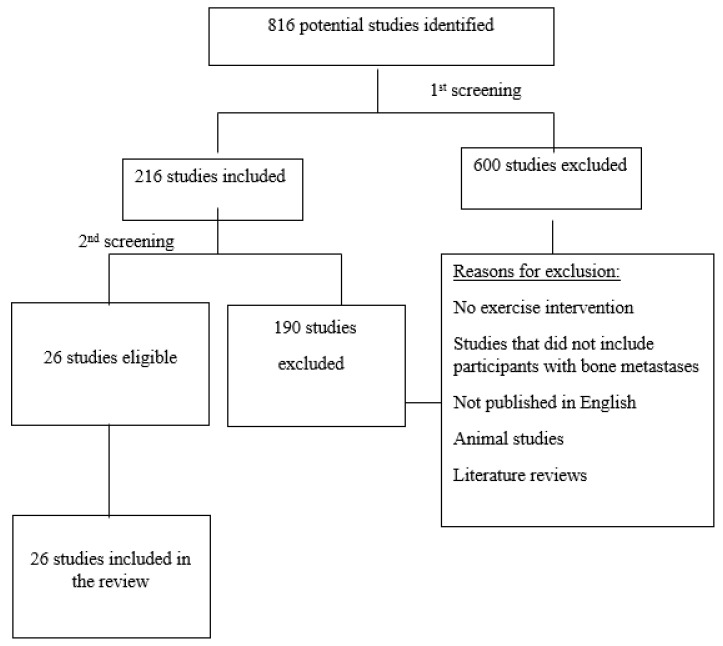
Workflow and selection process for document inclusion in the scoping review.

The reviewing process was conducted by the team to ensure there was a consensus on inclusion and exclusion criteria as shown in Supplementary Table S1. The search and selection process is shown in Figure 1. 

Stage 4: Charting the data. 

The data reported in eligible studies were charted in an Excel spreadsheet. Charted data included authors, year of publication, title, study type (e.g., clinical trial), recruitment period, population, type and stage of cancer, aims of the study, overview of methods, type and duration of the exercise intervention, in-person or remote supervision, outcome measures, use of assisted technology, participant compliance and adherence, results and skeletal-related events. 

Stage 5: Collating, summarizing and reporting the results. 

The 26 included studies were analysed for: (1) all numerical results reported; (2) the results specifically addressing our research question; and (3) implications for future research and practice. 

### 2.2. Eligibility Criteria

The PICOS approach (Population, Intervention, Comparator, Outcomes and Study design) was used to examine the studies for eligibility, as detailed in the next sections. 

#### 2.2.1. Population

The study population for this review was men or women of any ethnicity or setting with bone metastases. Only studies that included a majority of adults were selected (at least 80% of the sample was aged 18 years or older). This is a common criterion [6] and the rationale is that the paediatric population may confer different findings due to incomplete and ongoing bone development [7]. Studies that did not include any participants with bone metastases were excluded from this review. 

#### 2.2.2. Intervention

The included studies had an exposure group composed of individuals with bone metastases who were treated with an exercise intervention. For the collective data set, data were pooled for exercise vs. non-exercise (control) participants. 

#### 2.2.3. Comparator

Studies were included when the comparison group was composed of individuals with cancer who did not receive exercise intervention (a control group treated with standard care). 

### 2.3. Information Sources

Online databases were used to identify papers published between 2000 and 2022. PubMed, Scopus, NIH Clinical Trials and Google Scholar were searched until the 30 October 2022. 

### 2.4. Search Strategies

Eligible studies were identified by searching the above-mentioned electronic databases and then scanning the reference lists of selected studies for additional relevant documents.

Highly sensitive filters were used on PubMed, Scopus, NIH Clinical Trials and Google Scholar. Restrictions were imposed on publication date, publication status and language. Results from database searches were collated and duplicates were manually removed using Endnote reference software version X9.3.3 when the title, authors, study and year of publication were identical. Errata, retraction and expression of concern were checked. 

### 2.5. Selection of Sources of Evidence

To ensure consistency in the decision-making process, both reviewers screened the same 26 publications, discussed the results and evaluated the titles, abstracts and full texts of all studies.

### 2.6. Data Charting Process

Data from eligible studies were charted using a data extraction tool designed for this review. The tool captured the relevant information on key study characteristics and detailed information on all metrics used to evaluate exercise intervention anywhere in the article, including metrics that were not mentioned in the narrative explicitly. 

The reviewers discussed the charted results as a team. Disagreement was resolved by re-examination of the objectives of the review. 

### 2.7. Synthesis of Results

Studies were grouped by the method of exercise delivery: remote, in-person or mixed supervision. The study settings, participant characteristics, study design and outcome measures were analysed and reported.

## 3. Results

### 3.1. Selection of Sources of Evidence

A total of 816 citations were identified from the selected electronic databases. Based on the title and abstracts, 600 studies were excluded, and the remaining 216 full-text articles were retrieved and assessed for eligibility. Of these, 190 studies were excluded for the following reasons: studies that did not include an exercise intervention, studies that did not include participants with bone metastases, studies that are literature reviews, animal studies and studies that were not published in English. The elimination of duplicates by Endnote resulted in 155 articles. 

The second review of full-text articles resulted in a total of 26 studies that were considered eligible for this review (Supplementary Table S1). The workflow and selection process are detailed in Figure 1. 

### 3.2. Characteristics of Sources of Evidence

**Figure 2:**
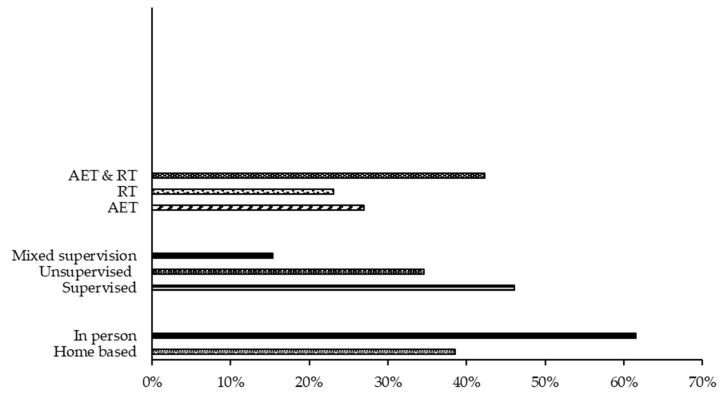
Characteristics of the exercise interventions used in reviewed studied (%). Abbreviations: AET, aerobic training; RT, resistance training.

Of the 26 included trials, one trial was a non-randomised clinical trial [8] and the remaining 25 were randomised controlled trials (RCT) [9,10,11,12,13,14,15,16,17,18,19,20,21,22,23,24,25,26,27,28,29,30,31,32,33]. Twelve trials (46.2%) assessed only participants with bone metastases; the remaining fourteen (53.8%) included both participants with and without bone metastases. A total of 1777 participants were included in the present review; of which 1319 participants (74.2%) had bone metastases, and 1132 participants (63.7%) were allocated to an exercise intervention (Figure 2). Seventeen trials (65.4%) included participants with prostate cancer [8,9,10,11,13,14,15,17,20,21,22,23,24,26,30,31,32]; 11 trials (42.3%) included participants with breast cancer [8,10,12,14,17,18,19,20,22,27,34]. Trials that recruited participants with mixed tumours (including breast and prostate) accounted for 65.4% (17 trials) [8,10,14,17,20,22,25,28,29,32,34] (Figure 2). 

### 3.3. Intervention Characteristics 

The characteristics of the exercise interventions are shown in Figure 2. Eighteen trials (69.2%) prescribed resistance training [8,9,10,11,12,14,15,16,17,21,22,24,25,26,29,31,32,33,34] (6 trials were resistance training only [8,11,14,26,32,33]); 12 trials included both resistance and aerobic exercise [9,10,12,15,16,17,21,22,24,25,29,31]). There were six trials (23.1%) that implemented aerobic exercise training only [13,17,18,19,20,30]. Resistance training specifically targeting spinal metastases was used in three trials (11.5%) [14,32,34], whereas a whole body approach was the focus of the remaining 14 trials that included resistance training. 

This study classified supervised exercise intervention as in-person, face-to-face with qualified personnel, whereas unsupervised exercise was carried out by participants on their own. Mixed supervision involved initial or partial sessions with qualified personnel followed by unsupervised exercise. Supervised (in-person) exercise intervention was used in 13 trials, accounting for 46.1% [8,10,11,14,15,17,18,22,26,27,29,30,32]. Nine trials (34.6%) were implemented without supervision [9,19,20,21,23,25,28,31,35]. A mixed approach to supervision was reported in four trials (15.4%) [12,24,32,33]. A total of 518 participants (29.2%) had no supervision during their exercise intervention. All supervision of exercise, regardless of mode of delivery, was carried out by trained personnel.

### 3.4. Safety of Exercise Intervention

Out of 26 studies included in this review, only 2 trials [13,30] (7.7%) reported serious adverse events: 2 fibula fractures and 1 partial Achilles tendon rupture. Both of these trials [13,30] were supervised in-person. The remaining 24 (92.3%) exercise interventions reported no serious adverse events [8,9,10,11,12,14,15,16,17,18,19,20,21,22,23,24,25,26,27,28,29,31,32,33]. It is noteworthy that of the 12 trials that were exclusively designed for participants with bone metastases, no skeletal related events were reported [8,9,14,15,16,17,18,20,22,23,32,34]. 

**Figure 3:**
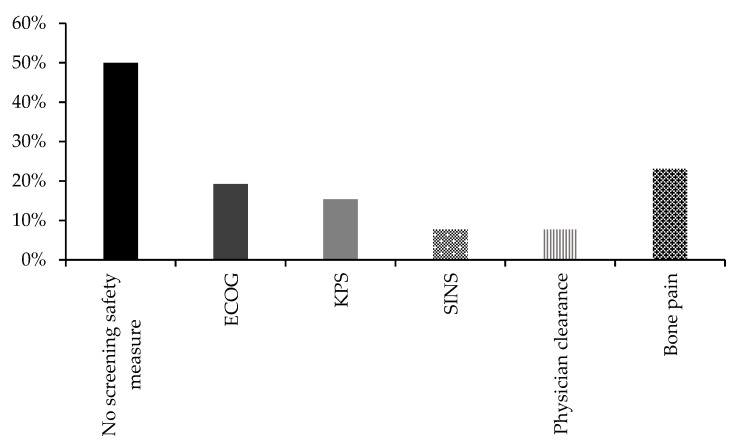
Safety screening in the included trials, presented as percentage. Abbreviations: ECOG, Eastern Cooperative Oncology Group; KPS, Karnofsky Performance Status; SINS, Spinal Instability Neoplastic Score.

Safety measures for recruitment of participants were not defined in 13 (50%) of the trials [9,13,14,16,17,18,19,20,21,23,25,27,33], whereas 13 trials had screened participants for ambulatory and self-care ability (Eastern Cooperative Oncology Group, ECOP) (5, 19.2%) and performance status (Karnofsky Performance Status, KPS) (4, 15.4%) [8,12,22,26,28,29,31,32,34] (Figure 3). Two trials (7.7%) used Spinal Instability Neoplastic Score (SINS) and the prognosis score Mizumoto [14,32]. Two trials required clearance from participants’ physicians [10,26]. Bone pain was assessed in six trials (23.1%) [11,12,15,24,29,30] (Figure 3). 

### 3.5. Intervention Efficacy

**Figure 4:**
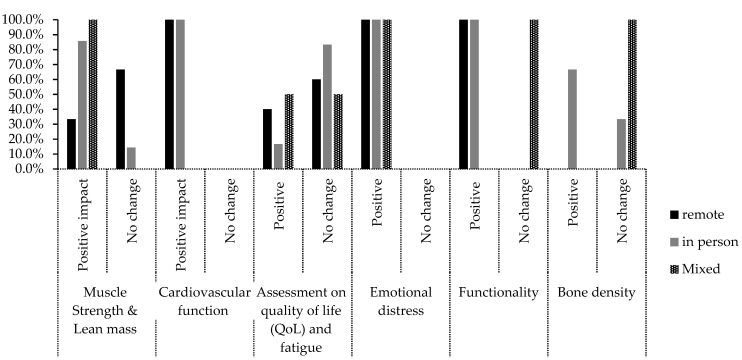
Patient outcomes displayed as percent (%) by trial delivery for: muscle strength, cardiovascular function, QoL, emotional distress, functionality and bone density.

None of the 26 trials included in the review reported serious negative effects of exercise intervention. Twenty-five trials reported an average compliance to the program of about 81% (58% to 100%), standard deviation of 14.1% with exercise well tolerated (Supplementary Table S1). Functional ability of patients was assessed in ten trials with nine (34.6%) reporting improvement [11,15,17,18,21,23,25,27,34] and one reporting no improvement [26] (Figure 4).

Muscle strength and lean mass were reported in eleven trials with eight trials reporting positive outcomes [8,11,13,16,17,26,30,34] (six using in-person training, one which used remote training and one which used mixed method training). In three of the trials [15,28,31], there was no change in muscle strength (two remote training trials and one in person trial). 

Both trials that measured cardiovascular function [9,10] reported improvement (one remote and one in-person trial) (Figure 4). 

Quality of life (QoL) and fatigue remained unchanged in nine trials (three remote, five in-person, and one mixed method trial) [8,11,13,15,17,20,29,31,32], but was improved in four trials (two remote, one in-person and one mixed method trial) [18,25,26]. Emotional distress was improved with exercise intervention in five trials (two remote, two in-person and one mixed method trial) [13,16,23,24,34]. For bone density, two trials reported an increase [13,36] (both used in-person training), and two trials reported no change [14,32] (one in-person trial, and one that used mixed in-person and remote training) (Figure 4). 

**Figure 5:**
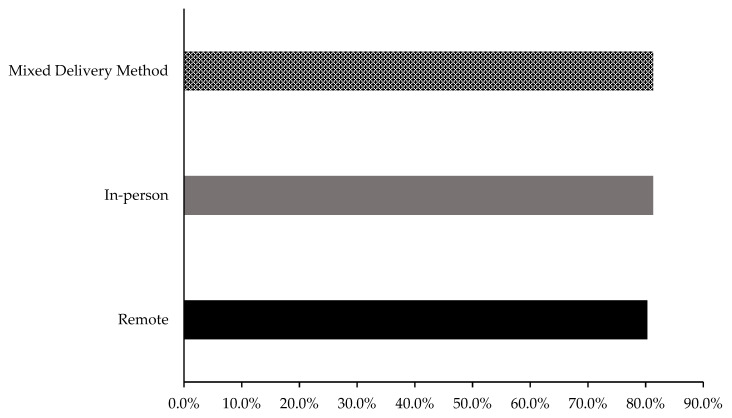
Participant compliance to the exercise intervention in remote, in-person and mixed delivery studies.

Remote delivery exercise intervention had an average of 80.3% compliance [9,12,16,19,20,21,23,25,28], whereas in-person [8,10,11,13,14,15,17,18,22,27,29,30,37] and mixed delivery methods [24,32,33] both had an average of 81.3% compliance (Figure 5). 

## 4. Discussion

The key findings from this scoping review are that exercise adherence was similar between in-person supervised trials and remotely delivered trials and that exercise was safe for cancer patients with bone metastases across all trials. This is an exciting finding, as it supports the notion that exercise medicine can be provided to cancer patients remotely, safely and effectively, expanding the opportunity for participation to those with limited access due to physical, geographical or social disadvantage and to those with a preference for home-based activity. 

Research in exercise for cancer patients has led to tremendous progress in treatment and survival over the last few decades. Since the pioneering study in the 1980s by Drs. Winningham and MacVicar, who conducted a randomized trial to investigate the efficacy of aerobic exercise on a group of women with breast cancer [38], the perception of exercise for cancer patients has moved from controversial to necessary. Exercise is now recognized as an important adjuvant therapy for cancer patients, having a significant beneficial impact on both disease and patient outcomes [1,39]. Despite fear of causing skeletal complications, corroborative evidence indicates that exercise/physical activity should be included in the treatment regime of cancer patients with bone metastases [1,2,3,38,39]. This scoping review supports the findings of these studies, reaffirming that exercise is safe and feasible for cancer patients with bone metastases. 

Some studies included in this review carried out safety screening before conducting the trials. Different measures were implemented, such as the KPS, ECOP, Spinal Instability Neoplastic Score (SINS) and the prognosis score Mizumoto, and assessment of bone pain. Of note, only six of the 26 studies assessed bone pain [11,15,17,22,32,33]. As bone pain is the most common complication of bone metastasis [35] and impedes exercise, it should be considered as a standard tool for screening.

Of the 26 studies included (1132 participants with bone metastases undertaking exercise intervention), there were only three adverse events reported. Two tibial fractures and one tendon rupture which occurred during the prescribed soccer training. Of the two fractures that occurred, both in the Bjerre et al. (2019) study [13], one participant belonged to the usual care group (non-exercise control) and the other to the soccer training group. Importantly, no bone metastases were evident at the fracture sites; thus, these injuries were most likely unrelated to metastatic disease. Despite various levels of efficacy, the general consensus is that exercise helps improve functional ability, muscle strength, lean mass and cardiovascular function. 

Expert consensus has demonstrated less than half of physicians and nurse practitioners felt confident to recommend exercise to people with bone metastasis [1]. Concerns over the possibility of causing skeletal complications are likely the reason for this conservative approach [1]. Encouragingly, the results from this review indicate no correlation between exercise prescription and serious adverse events [8,9,10,11,12,13,14,15,17,18,19,20,21,22,23,24,25,26,27,28,29,30,31,33]. Aerobic and resistance training, in particular, did not increase fracture risk or decrease functional ability, nor did it worsen fatigue or emotional distress. Considering the overarching recommendation by expert consensus that “the perceived risk of skeletal complication should be weighed against the potential benefits” [1], this review reaffirms that exercise intervention is indeed beneficial and could prevent further loss of functional capacity. The highlighted emerging challenge now is to improve patient access to individualised exercise treatment plans. 

In-person supervision was used in almost half of the trials included in this review [8,10,11,14,15,17,18,22,26,27,29,30,32] (46.1%), while mixed supervision [12,24,33] accounted for 15.4%. All supervision was carried out by trained personnel. This indicates general agreement with expert consensus of having qualified professionals with additional cancer-specific exercise training and adequate experience working with cancer patients [1]. Surprisingly, 34.6% trials were unsupervised [9,13,16,19,20,21,23,28,31], of which most were implemented in the last five years. The recency of this move toward unsupervised exercise may reflect uptake of internet and telephone health services during COVID-19, but may also indicate an empowerment strategy, giving patients more ownership and responsibility over their treatment. 

The results from this review support remote delivery of exercise intervention. Over one-third (38.5%) of trials did not have supervision for their exercise treatment, except for the initial consultation/demonstration of exercise [9,13,16,19,20,21,23,28,31]. Importantly, no serious adverse events were reported in unsupervised trials. This encouraging finding has significant meaning to patients who encounter difficulties attending in-person therapy due to physical, psychological or environmental barriers [40]. Removing these barriers and empowering patients to manage their exercise in their time and at their convenience could increase access to individual targeted exercise treatment. The question of whether remote-based delivery [9,12,16,19,20,21,23,25,28] is adhered to was also answered in the finding of 80.3% compliance, which rivals the 81.3% of in-person training [8,10,11,13,14,15,17,18,22,27,29,30]. It is suggested that remote-based delivery is safe and patient adherence is high, provided there is initial instructions from trained personnel. The included studies also implemented telephone calls for check-up and to motivate participants to comply with the exercise plan [9,12,16,21,25,28,31]. Overall, remote-based exercise appears to be a promising avenue to be explored by healthcare professionals. 

This review considered several patient outcome measures when comparing the efficacy of remote and in-person exercise delivery methods, including muscle strength, cardiovascular function, QoL, emotional distress, functional ability and bone density. The in-person approach improved participants’ muscle strength and lean mass more than the remote [16] and mixed delivery methods [26], with seven out of nine trials reporting positive outcomes [8,11,13,17,30,33,34]. Cardiovascular function was only assessed in one remote [9] and one in-person trial [10], both which reported an increase in cardiorespiratory fitness and improved performance on a short physical test battery. Quality of life (QoL) was improved in two remote trials [16,25], one in-person trial [18] and one mixed delivery trial [26]. This result was positive, though the majority of trials reported no change in this patient-reported outcome: three remote-based trials [19,29,31], five in-person trials [8,11,13,15,17] and one mixed delivery trial [32]. Arguably, QoL should be a high priority when treating patients with bone metastases, particularly when implementing an exercise intervention. 

Psychological support is an integral component of quality care for cancer patients [41]. Aligning with this, exercise therapy was beneficial for psychological factors in two remote based trials [16,23], two in-person trials [13,14], and a mixed delivery trial [24]. Participants in these trials reported improvements in social function, mental health and vitality. Bjerre et al. (2019) attributed this finding to peer support and greater control given to participants regarding their own health outcomes [13]. 

Functional ability refers to the capacity of participants to complete activities required for independent living, and it is enhanced with aerobic fitness [42]. Improvement was reported in three remote-based [21,23,25] and six in-person trials [11,15,17,18,27,33]. Participants experienced greater self-rated physical function, walking capacity, performance on the timed up and go test [11,17,23,27], submaximal exercise capacity and ambulation [11,33]. These positive results suggest that exercise therapy was important for patient independence [43]. 

Bone density was only measured in two in-person trials [13,36]. These studies showed increased hip bone mineral density (BMD) attributed to impact loading [13] and resistance training [36]. These findings were consistent with other studies, adding to the evidence that strength training may improve bone loss [44,45,46]. Winters-Stone et al. (2011) reported a significant increase in spine BMD and osteocalcin [44] and Vehmanen et al. (2021) claimed that exercise could potentially maintain femoral neck and total hip area BMD for up to three years [46]. Results from two other trials [8,32] are in opposition, with Rosenberger et al. (2017) arguing that proof of a positive effect was missing [8]. Sprave et al. (2019) also reported no improvement [32] in BMD. Further research is needed to confirm the effect of exercise on bone density for cancer patients.

Patient outcomes, particularly increases in muscle strength and lean mass, compared between modes of exercise delivery tend to favour in-person exercise, but it is important to consider that there were a greater number of in-person studies and each study reported limitations. Furthermore, with different patient outcome measures across the various studies, it is challenging to make a conclusion about the superiority of delivery methods. It is, however, promising for patient freedom of choice and accessibility that both delivery methods had similar compliance and no reported adverse effects. 

This scoping review included randomised controlled trials with high internal validity and a large number of participants, 1319 (74.2%), with bone metastases. This is a strength with regard to interpretation of the results. A notable limitation lies in the lack of detail in the reporting of bone metastasis characteristics (site, treatment, pharmacological history) which could influence the application of exercise into clinical practice. 

## 5. Conclusions

This review examines the existing knowledge on exercise intervention for patients with bone metastases. It appears that with initial coaching by qualified personnel and ongoing communication, exercise therapy for bone metastases patients can be delivered in-person or remotely, while being safe and effective, thereby enhancing access.

## Supplementary Materials

The following supporting information can be downloaded at: https://www.mdpi.com/article/10.3390/cancers15061786/s1, Table S1: Summary table of key characteristics of articles included in the scoping review.
