# Supplementary material for: Exercise Intervention for Bone Metastasis: Safety, Efficacy and Method of Delivery
Source: Cancers (Basel). 2023 Mar 15;15(6):1786. doi: 10.3390/cancers15061786 (PMC10046160; doi:10.3390/cancers15061786)
Supplement: Supplementary file 1 [file cancers-15-01786-s001.zip › cancers-2171025-supplementary.pdf]

Study type	Recruit-ment Pe-riod	Popula-tion	Type of cancer	Stage of cancer	Aim of the study	Overview of methods	Type of exercise	Duration of exer-cise	Other adju-vant thera-pies	Refer-ence
experi-mental	4 weeks	32 males	Prostate	metasta-sised	Assess feasibility of exercise for pa-tients with meta-static cancer	intervention	aerobic and re-sistance	12 week	androgen depriva-tion therapy ADT	[9]
experi-mental	25 months	66 males and fe-males	breast, lung, col-orectal. prostate, gyneco-logic, lym-phoma	advanced with 16 has bone metastasis	To compare the ef-fects of resistance and cardiovascular exercise on func-tional mobility in individuals with advanced cancer.	Prospective, 2-group pre-test-posttest pilot	re-sistance, cardio-vasucl ar,	10 weeks of indi-vidualised exercise	chemo-therap y, radiation	[10]
experi-mental	12 months	20 males	Prostate	metastasis	feasibility trial was to determine the safety and efficacy of resistance exer-cise by prostate cancer survivors with bone meta-static disease.	intervention, two armed prospective controlled trial	re-sistance exercise	twice weekly for 12 weeks	androgen depriva-tion therapy ADT, radiation	[11]
experi-mental	9 months	14 fe-males	breast	metastasis	determine the safety and feasibil-ity of a physical activity program for women with metastatic breast cancer ; explore the effi-cacy of the pro-gram.	pilot random-ized con-trolled trial	re-sistance exercise & brisk walking	8 weeks, 10-15 mins brisk walk fol-lowed by 30-40 mins of resistance training	hormone therapy, chemo-therap y	[12]
experi-mental	6 months	60 males and fe-males	lung, breast, prostate, mela-noma, kidney, others	metastasis	Compare the ef-fects of RT versus passive physical therapy on QoL, fa-tigue, and emo-tional distress out-comes during radi-ation therapy in patients with spinal bone metastases under radiotherapy (RT).	randomised, controlled, ex-plorative in-tervention trial	re-sistance training vs phys-ical therapy (in form of breathing exercise)	resistance training 30 mins; physical therapy 15 mins; three times/wee k for 6 months	hormone therapy, chemo-therap y, immuno-thera py,	[33]
experi-mental	6 months	60 males and fe-males	lung, breast, prostate, mela-noma, kidney, others	metastasis	To compare the ef-fects of RT versus passive physical therapy on bone density during ra-diation therapy (RT) in patients with spinal bone metastases. To quantify pathologi-cal fractures	randomised, controlled, ex-plorative in-tervention trial	re-sistance training vs pas-sive physical therapy	6 months, resitance training 30 mins, passive physical therapy 15 mins.	hormon-other apy, im-muno-thera py, chemo-therap y, radio-therapy, orthope-dic corset	[33]
					to explore whether patients with			6 months, treated		

experi- mental	21 months	214 males	prostate	various in- cluding bone me- tastasis (21%)	prostate cancer ac- cepted exercise in a local football club under regular con- ditions, and to ex- amine the potential effects of 1 year of community based football training in terms of both ran- domised partici- pants and just those adhering to the protocol.	2 armed, mul- ticentre ran- domised con- trolled trial	playing football	group - twice a week for an hour; control group 15- 30 min phone ses- sion re options for physi- cal activ- ity and free rehab	radiation, chemo- therap y, anti- androgen therapy	[13]
experi- mental	not men- tioned	60 males and fe- males	lung, breast, prostate, others	spinal me- tastasis	to investigate the feasibility of free isometric spinal stabilization exer- cises concomitant to palliative- anal- getic radiotherapy in patients with un- stable spinal me- tastases	exploratory randomized controlled fea- sibility tiral	free spi- nal sta- bilization exercise on the day of radio- therapy (2 weeks) and then home- based for 3 months; CT - progres- sive muscle relaxa- tion ex- ercise daily on 9+- 2 days with radio- therapy : read or played from re- cordings 15mins a day on upper and lower extrem- ity	3 months	medica- tion, ra- diation	[14]
experi- mental	36 months	57 males	Prostate	bone me- tastasis	Test the efficacy and safety of a modular multi- modal exercise program (M3EP) comprising re- sistance, aerobic, and flexibility	two armed prospective randomized controlled trial	M3EP: re- sistance, aerobicm flexibil- ity exer- cises, 3 times/we	3 months	ADT, radiation, brachy- therap y, chemo- therap y	[15]

				training to maintain or enhance physical function		ek, 60mins a session			
experimental	24 males and females	MM	MM with bone lesions	to test the suitability of home-based exercise therapy for patients undergoing high- dose chemotherapy and autologous peripheral stem cell transplantation.	feasibility/pilot study	aerobic component (usually walking, but sometimes running or cycling depending on fitness and desires of patients) and strength resistance training (using exercise stretch bands)	6 months		[16]
experimental	20 males and females	Breast or prostate	bone metastasis	determine whether a 3- month supervised resistance exercise program results in any sustained functional benefits in prostate and breast cancer patients with bone metastatic disease	pilot randomized controlled trial	twice weekly resistance exercise sessions, 60 mins/session ; participants were encouraged to undertake home-based aerobic exercise sessions of walking and/or stationary cycling, with the aim of accumulatin g a total of 150 min	3 months	hormone, radiation, chemotherap y, surgery	[17]

							of moderate intensity aerobic exercise each week			
experimental		1 female	breast	bone metastasis	determine the feasibility and effects of an aerobic exercise program for patients with bone metastases.	feasibility/pilot study	ergometer cycling, 3 times a week. 30 min at the beginning to 50 min after 2 weeks.	1 year	three cytotoxic agents and pamidronate and radiotherapy	[18]
experimental		49 females	breast	bone metastasis - 67%	assess physical activity preferences before and after a 6-month intervention to investigate demographic and clinical correlates of these preferences	single arm trial	150 min per week of moderate-to-vigorous physical activity, including 30-min walking sessions per day	6 months	hormone therapy, chemotherap y	[19]
experimental	9 months	12 males and females	breast, prostate, lungs, colon	bone metastasis	feasibility of using activity trackers (AT) to assess the patient prognosis and the effects of palliative RT	pilot clinical trial	walking	7 days post RT	radiotherapy	[20]
experimental	12 months	40 males	Prostate	metastasis, over 80% with more than one bone metastasis	acceptability, safety and preliminary efficacy of webbased exrecise intervention ExerciseGuide	pilot, randomised, two armed, controlled	erobic, resistance-based and flexibility exercise prescription based on info provided by patients	8 weeks	radiotherapy, chemotherap y, hormone therapy	[21]
experimental		48 males and females	various	secondary bone cancer	determine the risk of PF following physical rehabilitation in people with secondary bone cancer	Single-group, single- centered interventional clinical trial	aerobic, anaerobic, and flexibility training	8 months	NA	[22]

experimental	3 years	55 males	prostate	bone metastasis	the association between physical activity levels and physical and mental health outcomes in prostate cancer patients with bone metastases	Cross sectional intervention, randomised	various	3 months	radiation, chemotherap y, hormone therapy	[23]
experimental		100 males	prostate	locally advanced or metastatic	evaluate the effect of a lifestyle intervention on disease-specific QoL, diastolic blood pressure, and cancer-related fatigue in sedentary men receiving longterm ADT for advanced prostate cancer	randomised, controlled, two armed single blind trial	aerobic and resistance exercise with parallel dietary advice	12 weeks	ADT	[24]
experimental	3.5 years	516 males and females	solid or hematologic cancer	stage IIIC or IV	determine whether collaborative telerehabilitation and pharmacological pain management improve function, lessen pain, and reduce requirements for inpatient care.	3-arm randomized clinical trial	walking, resistive exercise program, pharmacolog ical intervention	6 months	NA	[25]
experimental	10 months	37 males	prostate	various including bone metastasis (37.5% for exercise group; 36.8% for non exercise group)	the effect of resistance training on body composition and metabolic syndrome MetS changes in prostate cancer patients on ADT; the effect of exercise on sarcopenia prevalence, body fat, strength, physical function, quality of life, and cardiometabolic markers, including insulin, insulin resistance, and the MetS components of blood pressure, central adiposity, triglycerides, glucose and HDL-C	4 armed randomised, explorative	resistance training	12 weeks	ADT	[26]
							aerobic trainign or stretching, treadmill walking ses- sions delivered thrice weekly between 55% and			

experimental		65 females	breast	Stage IV metastatic	determined the feasibility and safety of aerobic training in metastatic breast cancer, explore the effects on symptom control outcomes and to identify a subgroup of patients for whom aerobic training was feasible	randomized 2 armed	80% of peak oxygen consumption (VO 2peak) for 12 consecutive weeks. Stretching was matched to aerobic training with respect to location, frequency, duration, and intervention length.	12 weeks	chemotherapy	[27]
experimental	2.5 years	46 males and females	non-small cell lung cancer or inoperable pancreatic cancer	stage III/IV	examine the feasibility and safety of a multimodal intervention (n-3 polyunsaturated fatty acid nutritional supplements, exercise, and anti- inflammatory medication: celecoxib) for cancer cachexia in patients with incurable lung or pancreatic cancer, undergoing chemotherapy; exploratory to inform future trial design, should the primary endpoints be positive and future trials be deemed worthwhile.	Phase 2, randomised, two armed	aerobic and resitance	6 weeks	surgery, chemotherapy, radiotherapy,	[28]

experi-mental	2.5 years	and fe-males	metastatic or locally advanced tumors of the gas-trointes-tina l, lung	Stage IV metastatic or locally advanced	test the effects of a combined nutrition and physical exer-cise program on cancer patients with metastatic or locally advanced tumors of the gas-trointestinal and lung tracts	two armed, randomized controlled trial	60-min ex-ercise program twice a week: warm-up exer-cises, strength and bal-ance training exercises	3 months	NA	[29]
experi-mental	NA	57 males	Prostate	advanced or locally advanced Pca, bone metastasis 24.1% in EX group, 15.4% in CON group	effects of 12 weeks football training on the LBM (primary outcome), body fat percentage, maxi-mal oxygen uptake (VO2max), muscle strength, and sit-to-stand performance	two armed, randomized controlled trial	football practice, 2-3 times a week	12 weeks	ADT	[30]
experi-mental	2 years	46 males	prostate	locally ad-vanced or advanced stage, 43% in EX group, 26% in CON group	the effects of 12 weeks of unsuper-vised home-based 'exergaming' (i.e., technology-driven exercise) com-pared to usual care on physical func-tion, body compo-sition, quality of life (QoL), and fa-tigue in patients with prostate can-cer on androgen-deprivation ther-apy (ADT)	two armed, randomized controlled trial	90 min individ-ual be-fore the home-based exergam-ing with the Xbox 360 Ki-nect sys-tem, aer-obic and strength exercise for 1 h, includ-ing a warm-up and cool-down period, three-times a week for 12 weeks	12 weeks	ADT	[31]
	2 years		various		to evaluate the safety/feasibility of IPMT and second-arily assess pain, bone density,	exploratory, non- blinded,	once daily, starting on the first day of pallia-tive RT and con-tinuing for the entire RT pe-riod, 15	duration of RT and then 3 times a week for		[32]

experimental		60 males and females		Bone metastasis	pathologic fracture rate, and QOL	randomized controlled trial	min per day and consisted of isometric exercises	another 3 months	radiotherapy	
experimental	34 months	20 males and females	renal cell, gastrointestina l, pancreatic, liver	locally advanced or metastatic, 30% bone mets	feasibility and describing effects of a supportive 12-week progressive resistance training program in advanced cancer patients undergoing TKI therapy	non-randomized controlled clinical exercise intervention trial	machine-based resistance training, 2x/week	12 weeks	Tyrosine kinase inhibitor	[8]

**Outcome measures**	**Compliance**	**Adherence to program**	**In-person or Remote based**	**Supervision**	**Assisted training technology**	**Main results**	**Positive association exercise with reduced fracture risk**	**Reference**
body composition, QoL, cardiorespiratory, max lower body strength	70%	80.7% for walking and 85.3% for resistance training	home-based/remote based	unsupervised	resistance bands of 4 different elastic tension, activity tracker (Garmin vivosmart)	feasible for men with prostate cancer, cardiorespiratory and max lower body strength increased	Not mentioned	[9]
Functional mobility (SPPB); self-reported pain and fatigue.	78.8% (67.7% in resistance arm; 90.6% in cardivascular arm)	not mentioned	in-person	monitored by trained personnel	not mentioned	significant increase in SPPB total score (P<.001), increase in gait speed (P=.001), and reduction in fatigue (P=.05). cardio exercise participants - small improvement in SPPB total score than RT participants (F1,49=4.21, P=.045), the difference was not confirmed	Not mentioned	[10]
safety and tolerance of the exercise program, physical function, physical activity level, body composition, fatigue, quality of life and psychological distress	93%	83%	in-person	monitored by trained personnel	Neurocom Smart Balancemaster for balance,triaxial accelerometer activity monitor for physical activity level	No AE or SRE occurred during the supervised exercise sessions. Program well tolerated, high attendance (83%) and compliance rates (93%), participants exercise at an intensity within the target range perceived ∼exertion =13.8±1.5). muscle strength 11%∼; submaximal	not clear	[11]

						ae∼robic exercise capacity 5% an∼d ambulation 12%), phys∼ical activity level (24%) and lean mass (3%) differed significantly between groups following the intervention, with favorable changes in the exercise group compared with the usual care group. No significant between- group differences were observed for fatigue, quality of life or psychological distress.		
feasibility & safety, preliminary efficacy, fatigue, QoL, physical performance, physical activity	100%	Adherence to the resistance and walking components of the program was 100% and 25%, and 71% adhered to the intensity of walking.	home-based/remote-based	mixed: supervised resistance training by trained personnel and unsupervised walking	portable bar and resistance bands, weights, pedometers, dynamometer, physical activity monitor (Actiheart)	feasible and safe. The dose of the resistance training component was well tolerated and achievable in this population	not clear	[12]
QoL, fatigue, and emotional distress.	Treatment group 83.3% for 12 weeks, 60% for 24 weeks; control group 73% for 12 weeks and 60% for 24 weeks	not mentioned	mixed: in-person and home based	mixed: first with trained personnel for 2 weeks then home based for 6 months	not mentioned	Psychosocial aspects in resistance training group were significantly improved after three (p = 0.001); and six months (p = 0.010).Painful site and pain characteristics were without significant differences. Functional interferenc (p = 0.081). physical fatigue (p = 0.013), and interference with daily life (p = 0.006) . Emotional distress was lower after six months (p = 0.016).	not clear	[33]
bone density involved metastatic bone during radiation therapy, quantify	100%	not mentioned	mixed: in-person and home based	mixed: first with trained personnel for 2 weeks then home based for 6 months	not mentioned	Bone density increased by 28.3% (IQR 11.4–139.0) and 80.3% (IQR	yes	[33]

pathological fractures						32.6–250.6) after 3 and 6 months INT (both p < 0.01). compared to CT after 3 and 6 months (both p < 0.01, median 59.7; IQR 21.1–98.3 median 62.9; IQR 9.7 to 161.7). control group . 23.3% of the patients in the treated group and 30.0% of the patients in control group had pathological fractures, no fracture was assigned to intervention, and no difference between groups after 3 and 6 months was observed (p = 0.592 and p = 0.604)		
QoL, and secondary outcomes: continuation of football after 6 months, hip and lumbar spine BMD, mental health score, fat lean body mass, safety outcomes ie, fractures, falls and hospital admissions	95% at 6 months, 92% at 1 year	59% chose to continue after completion of intervetion and 78% attended more than 50%	in-person	self supervision	not mentioned	no difference in QoL, improved mental health & hip BMD, less fat mass INT, no difference in self-reported physical activity behaviour, higher hospital admissions in controlled group, no difference in fractures.	yes	[13]
feasibility, expressed as training attendance rate and training-related adverse events	90% for treatment group; 80% control group	around 50% could not do 2 out of 4 exercises	in-person	monitored by trained personnel	not mentioned	Attendance metrics high, high acceptance of the training program. frequent deviations from the training prescription: modified due to pain, immobility or weakness.	not clear	[14]
physical function subscale patient-rated, physical function by up & go test, dynamic muscle strength, balance, whole body lean and fat mass, fatique, prostate specific antigen, safety of program	89%	not mentioned	in-person	monitored by trained personnel	not mentioned	no difference for self- reported physical function (P = 0.682) at baseline. After 3 months, significant difference in physical function EX (3.2 points; 95% CI, 0.4–6.0 points; P =0.028). no diffence in	no fractures.	[15]

						baseline for leg extension muscle strength, physical function, body composition or fatigue. after 3 months, treatment group has improved leg extension.<br>No change in fatigue, physical function, balance, lean mass or total body fat mass. No adverse events of skeletal fractures		
	75%		homebased, many patients lived out of state, telephone contact to determine if modifications are needed	self supervision after initial test for exercise prescription	stretch bands	all 14 patients in INT improved in : lean body weight, muscle strength, fewer minutes on treadmill, decrease in fatigue, mood disturbance, increase night sleep, decrease daytime sleepiness, and the test results of all 10 patients in the usual- care group declined.	yes, no SREs.	[16]
Safety and tolerability of exercise, Physical function and physical activity levels, Body composition. Patient reported outcome	85%		in person	the supervision of an accredited exercise physiologist		14 participants completed the follow- up . improvements in physical function (4–6 %), physical activity levels (~160 min/week), lean mass (3–4 %), and QoL(5–7 %). 6-month , improvements in ambulation (4 %), physical activity level (~105 min/week), whole body lean mass (2 %), and quality of life (13 %) remained.	yes, no SREs.	[17]
HR, respiratory quotient, O2 uptake, peak work capacity, QoL, patient reported outcome	NA		in person	Supervised	cycling machine	Feasibility, safety and beneficial effects of the program were proven for the patient in this case study. VO 2max (20.2% after 16 weeks, 52.7% after 12 months) and peak work capacity (15.5% after 16 weeks, 35.7% after 12 months)	yes, no SREs.	[18]

	had increased. marked improvement in physical performance and in quality of life.	

Physical activity preferences	89%		remote based/home based	unsupervised	activity tracker	More participants preferred community fitness centre (66%) rather than at home (19% vs. 44% at baseline, p =.03). A higher social vulnerability score and no chemotherapy at baseline significantly associated with lower desire to receive physical activity counselling (p =.01 and p =.04 respectively).	NA	[19]
QoL, pain,	NA		remote/home based	unsupervised	activity tracker	No correlation between improvements in QoL or level of pain with number of daily steps. Patients who took more than 7800 steps per day prior to radiotherapy lived significantly longer	NA	[20]
acceptability, safety, efficacy, patient reported outcomes, trial feasibility	resistance:64.6+/- 40.2%; aerobic 102 +/- 62.7%	resistance training: 78.3+/- 77.9%; aerobic 91.5+/ 56.5%	remote/home based	unsupervised	Exercise-Guide	no serious adverse effect, higher moderate to vigorous physical activity, improvement in stepcount, ExerciseGuide is acceptable, safe and efficacious	No SREs	[21]
safety and feasibility of an outpatient rehabilitation program for patients with SBC; Short Form Health Survey (SF-36) and numeric rating scale (NRS) scores to assess pain intensity	58%	NA	in person	supervised	NA	One PF was detected, outside of rehabilitation therapy. Two other SREs occurred, a total event rate of 11.8/ 10 000 hours of exposure. significant improvement in NRS (95% CI, 1.41-3.08, P <.001) and SF-36 measures (95% CI, 80.35-158.11, P <.001)	No fractures	[22]
physical activity, QoL, physical performace, , body size/ composition and prostate-specific antigen	58% moderate and vigorous exercise; only 29% met the current aerobic execise for cancer survivors (>150 mins of moderate intensity or >75 mins of vigorous	29%	remote based	unsupervised	NA	16 (29%) met aerobic exercise guidelines, 39 (71%) lower aerobic exercise levels- lower physical functioning (p = .004), role functioning (physical and emotional) (p <	NA	[23]

	intensity per week)					.05), general health scores (p = .014) , lower physical performance (p < .05), reduced physical and mental health		
Exercise behaviour, adherence to the intervention, and biochemical safety; health-related outcomes, dietary outcomes	85% of the cohort completing 12-wk follow-up and 68% of men attending follow- up at 6 mo	94% for the supervised and 82% of the prescribed independent exercise sessions over the first 12 wk.	mixed: in-person and home based.	Supervision twice a week from weeks 1-6; once week 7-12; first 6 weeks, at least once a week self directed, increasing to twice a week from week 7-12	NA	Clinically relevant improvements in FACT- P were seen at 12 wk in the INT compared with CT (mean difference: 8.9 points; 95% confidence interval [CI], 3.7–14.2; adjusted p = 0.001). No difference was apparent at 6 mo (mean difference: 3.3 points; 95% CI, 2.6 to 9.3; adjusted p = 0.27). No difference in diastolic blood pressure was seen at either follow-up (all p > 0.05). Clinically relevant improvements in FACT-F were seen at 12 wk (mean difference: 5.3 points; 95% CI, 2.7–7.9; adjusted p < 0.001) and maintained following withdrawal of supervision (mean difference: 3.9 points; 95% CI, 1.1–6.8; adjusted p = 0.007).	NA	[24]
Blinded assessment of function using the Activity Measure for Postacute Care computer adaptive test, pain interference and average intensity using the Brief Pain Inventory, and quality of life using the EQ-5D-3L	97.5% at month 3 and 97.4% at month 6 and were similar across the arms	NA	home based	automated monitoring, telephone consultation with qualified PT	pedometer, elastic bands, supported documents	Compared with CT, the telerehabilitation arm 2 had improved function (difference, 1.3; 95% CI, 0.08-2.35; P = .03) and QoL (difference, 0.04; 95% CI, 0.004-0.071; P = .01), while both telerehabilitation arms 2 and 3 had reduced pain interference (arm 2, −0.4; 95% CI, −0.78 to		[25]

	−0.09; P = .01 and arm 3, −0.4; 95% CI, −0.79 to −0.10; P = .01), and average intensity (arm 2, −0.4; 95% CI, −0.78 to −0.07; P = .02 and arm 3, −0.5; 95% CI, −0.84 to −0.11; P = .006). Telerehabilitation was associated with higher odds of home discharge in arms 2 (odds ratio [OR], 4.3; 95% CI, 1.3-14.3; P = .02) and 3 (OR, 3.8; 95% CI, 1.1-12.4; P = .03) and fewer	

body composition, metabolic syndrome, QoL, physical fitness, muscle strength	Exercise group: 93.8 ± 2.0%with 77% of TRAIN and TRAINPRO participants completing all 36 sessions; Protein adherence: TRAIN-PRO and PRO groups was 72.0 ± 22.8%, with 120.9 ± 58.1 doses of 168 total ingested; Adherence to the home-based flexibility program in PRO and STRETCH was 79.0 ± 4.1%, with 28.4 ± 10.4 of 36 sessions completed	88.3 ± 16.0%	Exercise group: supervised exercise 3 days per week for 12 weeks; control; homebased	Exercise: supervised,		32 participated in the intervention (EXE n = 13; No-EXE n = 19). At baseline, 43.8% of participants were sarcopenic and 40.6% met the criteria for MetS. Post- intervention, EXE significantly improved lean mass (d = 0.9), sarcopenia prevalence (d = 0.8), body fat % (d = 1.1), strength (d = 0.8–3.0), and prostate cancer-specific quality of life (d = 0.9) compared to NoEXE ($p < 0.05$). No significant differences were observed between groups for physical function or MetS-related variables except waist circumference (d = 0.8).	No SREs	[26]
aerobic training feasibility, which was a priori defined as the lost to follow- up (LTF) rate (< 20%) and attendance (70%). Secondary end- points were safety, objective outcomes (VO2 peak and functional capacity, patient reported outcomes QoL	63% ±30%.	49%	in person	supervised		mean attendance rate was 63% ± 30%. permanent discontinuation and dose modification were 27% and 49%. improvements in PROs,favored the attention control group (P values > .05). 14 of 33 patients (42%) aerobic training had acceptable tolerability (relative dose intensity70%), improvements in VO2peak and functional capacity (P values< .05).	No SREs	[27]
		three patients had >80% compliance to all components of the intervention.						

feasibility and safety of a multimodal intervention (n-3 polyunsaturated fatty acid nutritional supplements, exercise, and anti- inflammatory medication; Weight, muscle mass, physical activity (ActivPAL and 6MWT), grip strength, nutritional status (aPG-SGA and AveS scores), and fatigue score	Compliance (deemed as >50% of individual components in 50% of patients) was 76% (19/25) for the celecoxib, 60% (15/25) for the exercise components and 48% (12/25) for the ONS. Therefore, acceptable compliance was achieved in all but the ONS.	In terms of combinations, eight (38%) patients did >80% of the aerobic and resistance components. Nine (43%) patients took >80% of the ONS and celecoxib components and nine (43%) patients took/did >80% of the resistance and celecoxib components.	home based	unsupervised, contacted once a week to assess compliance and to encourage adherence to the multimodal intervention	weights	Compliance to the individual components of the intervention was 76% for celecoxib, 60% for exercise, and 48% for nutritional supplements. no statistically significant effect on physical activity or muscle mass. no intervention-related SAE and survival was similar between the groups.	No SREs	[28]
Quality of life (European Organization for Research and Treatment of Cancer Quality of Life Questionnaire version 3.0), physical performance (hand- grip strength, 6-min walk test, timed sit- to-stand test and 1 repetition maximum leg press), nutritional status (body weight, bioelectrical impedance analysis), dietary intake (three- day dietary record) and clinical data (unexpected hospital days, performance status)	75%	NA	in person	supervised by qualified professionals	cycling ergome ter, machines for strength training, balance mat	Median adherence to exercise program 75%. The median number of counselling sessions was 3.0 (range 0–7 sessions). Post intervention, no difference in global health status/ QoL. Intervention was superior to UC regarding nausea and vomiting (p = 0.023) and protein intake (p = 0.01). No statistical differences were for energy intake, nutritional status and physical performance.	No SREs	[29]
						Mean heart rate during training was 137.7 (standard deviation 13.7) bpm or 84.6 (3.9)% HRmax. In FG, LBM increased by 0.5 kg [95% (CI)		

The primary outcome was change in lean body mass (LBM) assessed by dual-energy X-ray absorptiometry scanning. Secondary outcomes included changes in knee- extensor muscle strength (one repetition maximum), fat percentage, and maximal oxygen uptake (VO2max)	76.50%	NA	in person	supervised by qualified professionals	HR monitor	0.1–0.9; P = 0.02] with no change in CON (mean group difference 0.7 kg; 95% CI 0.1–1.2; P = 0.02). muscle strength increased in FG (8.9 kg; 95% CI 6.0–11.8; P < 0.001) with no change in CON (mean group difference 6.7 kg; 95% CI 2.8–10.7; P < 0.001). In FG, VO2max increased (1.0 mL/kg/min; 95% CI 0.2–1.9; P = 0.02) and fat percentage tended to decrease (0.7%; 95%CI 1.3–0.0; P = 0.06), but these changes were not significantly different from CON. In	Some SREs: 3 events, no bone metastasis present in the fractured bones --> accidental, unrelated to metastatic disease.	[30]

The primary outcome of the study was 6-min walking test (6MWT). Secondary outcomes were leg extensor power (LEP), body composition (lean- and fat-mass), self- reported physical functioning and global health status (European Organisation for Research and Treatment of Cancer quality of life questionnaire 30- item core [EORTC QLQ-C30]), QoL (Functional Assessment of Cancer Therapy – Prostate [FACT-P]) and fatigue (FACT – fatigue [FACT-F]).	91% of patients completing in the intervention group and 87% in the usual care group	NA	home based	unsupervised	Xbobox	significant improvement in the exergaming group compared to the CT in the primary outcome of 6MWT (mean difference: 21.5 m; 95% confidence interval ([CI]) 3.2–39.9; P = 0.023). no differences between the groups for LEP (P = 0.227), lean body mass (P = 0.100), fat body mass (P = 0.092), self-reported physical functioning (P = 0.084) and global health status (P = 0.113), QoL (P = 0.614), and fatigue (P = 0.147).	No SREs	[31]
feasibility (completion of training programs three months post- RT),pain response (Visual Analog Scale) and opioid consumption, bone density and pathologic fracture rate, and QOL (European Organization for Research and Treatment of Cancer, EORTC questionnaires)	≥80% of the planned sessions were completed by 55% (n = 16/29) in CON and 67% (n = 18/27) in INT	NA	in person during RT then another 3 month homebased	supervised by trained personnel	NA	no AE with either training regimen. Altogether, ≥80% of the planned sessions were completed by 55% (n = 16/29) in CON and 67% (n = 18/27) in INT. Regarding the post radiotherapy home- based training, ≥80% of planned sessions were completed by 64% (n = 9/14) of the INT cohort. There were no differences in pain scores, opioid consumption, or bone density between arms (p > 0.05 for all). No difference was observed between groups regarding new pathological	No SREs	[32]

						fractures (INT: n = 1 vs. CON: n = 3) after three months (p = 0.419). There were no QOL differences between arms (all parameters p > 0.05).		
feasibility through evaluating training attendance and completion rate, adverse events, and training progression. training-induced changes in fatigue, QoL, depression, and physical fitness	81%	unclear	in person	supervised by qualified professionals	exercise machine	beneficial effects on muscle strength (RT +11 ± 9 Nm, CON −13 ± 25 Nm, p = 0.005), but not on fatigue (RT +0.3 ± 4.1, CON - 1.5 ± 3.0, p = 0.223) or QoL (global QoL score RT −5.6 ± 16.1, CON −2.0 ± 18.2, p = 0.617).	No SREs	[8]

References

8. Rosenberger, F.; Wiskemann, J.; Vallet, S.; Haag, G.M.; Schembri, E.; Jäger, D.; Grüllich, C. Resistance Training as Supportive Measure in Advanced Cancer Patients Undergoing TKI Therapy-a Controlled Feasibility Trial. *Support. Care Cancer* **2017**, *25*, 3655–3664. https://doi.org/10.1007/s00520-017-3788-3.
9. Carver, J.L. Feasibility Study of a Home-Based Exercise Intervention Program for Patients with Metastatic Castration-Resistant Prostate Cancer Receiving Androgen—Deprivation Therapy; The University of North Carolina at Chapel Hill: Ann Arbor, MI, USA, 2018.
10. Litterini, A.J.; Fieler, V.K.; Cavanaugh, J.T.; Lee, J.Q. Differential Effects of Cardiovascular and Resistance Exercise on Functional Mobility in Individuals with Advanced Cancer: A Randomized Trial. *Arch. Phys. Med. Rehabil.* **2013**, *94*, 2329–2335. https://doi.org/10.1016/j.apmr.2013.06.008.
11. Cormie, P.; Newton, R.U.; Spry, N.; Joseph, D.; Taaffe, D.R.; Galvão, D.A. Safety and Efficacy of Resistance Exercise in Prostate Cancer Patients with Bone Metastases. *Prostate Cancer Prostatic Dis.* **2013**, *16*, 328–335. https://doi.org/10.1038/pcan.2013.22.
12. Yee, J.; Davis, G.M.; Hackett, D.; Beith, J.M.; Wilcken, N.; Currow, D.; Emery, J.; Phillips, J.; Martin, A.; Hui, R.; et al. Physical Activity for Symptom Management in Women With Metastatic Breast Cancer: A Randomized Feasibility Trial on Physical Activity and Breast Metastases. *J. Pain Symptom Manag.* **2019**, *58*, 929–939. https://doi.org/10.1016/j.jpainsymman.2019.07.022.
13. Bjerre, E.D.; Petersen, T.H.; Jørgensen, A.B.; Johansen, C.; Krustrup, P.; Langdahl, B.; Poulsen, M.H.; Madsen, S.S.; Østergren, P.B.; Borre, M.; et al. Community-Based Football in Men with Prostate Cancer: 1-Year Follow-up on a Pragmatic, Multicentre Randomised Controlled Trial. *PLoS Med.* **2019**, *16*, e1002936. https://doi.org/10.1371/journal.pmed.1002936.
14. Rosenberger, F.; Sprave, T.; Clauss, D.; Hoffmann, P.; Welzel, T.; Debus, J.; Rief, H.; Wiskemann, J. Spinal Stabilization Exercises for Cancer Patients with Spinal Metastases of High Fracture Risk: Feasibility of the DISPO-II Training Program. *Cancers* **2021**, *13*, 201.
15. Galvão, D.A.; Taaffe, D.R.; Spry, N.; Cormie, P.; Joseph, D.; Chambers, S.K.; Chee, R.; Peddle-McIntyre, C.J.; Hart, N.H.; Baumann, F.T.; et al. Exercise Preserves Physical Function in Prostate Cancer Patients with Bone Metastases. *Med. Sci. Sports Exerc.* **2018**, *50*, 393–399. https://doi.org/10.1249/MSS.0000000000001454.
16. Coleman, E.A.; Hall-Barrow, J.; Coon, S.; Stewart, C.B. Facilitating Exercise Adherence for Patients with Multiple Myeloma. *Clin. J. Oncol. Nurs.* **2003**, *7*, 529–534. https://doi.org/10.1188/03.CJON.529-534.
17. Cormie, P.; Galvão, D.A.; Spry, N.; Joseph, D.; Taaffe, D.R.; Newton, R.U. Functional Benefits Are Sustained after a Program of Supervised Resistance Exercise in Cancer Patients with Bone Metastases: Longitudinal Results of a Pilot Study. *Support. Care Cancer* **2014**, *22*, 1537–1548. https://doi.org/10.1007/s00520-013-2103-1.

18. Crevenna, R.; Schmidinger, M.; Keilani, M.; Nuhr, M.; Fialka-Moser, V.; Zettinig, G.; Quittan, M. Aerobic Exercise for a Patient Suffering from Metastatic Bone Disease. *Support. Care Cancer* **2003**, *11*, 120–122. https://doi.org/10.1007/s00520-002-0400-1.
19. Delrieu, L.; Vallance, J.K.; Morelle, M.; Fervers, B.; Pialoux, V.; Friedenreich, C.; Dufresne, A.; Bachelot, T.; Heudel, P.-E.; Trédan, O.; et al. Physical Activity Preferences before and after Participation in a 6-Month Physical Activity Intervention among Women with Metastatic Breast Cancer. *Eur. J. Cancer Care* **2020**, *29*, e13169. https://doi.org/10.1111/ecc.13169.
20. Dorion, V.; Lambert, L.; Frazzi, A.; Cayer, J.-F.; Wong, P. A Pilot Study in the Use of Activity Trackers for Assessing Response to Palliative Radiotherapy. *Cureus* **2017**, *9*, e1871. https://doi.org/10.7759/cureus.1871.
21. Evans, H.E.L.; Galvão, D.A.; Forbes, C.C.; Girard, D.; Vandelanotte, C.; Newton, R.U.; Vincent, A.D.; Wittert, G.; Kichenadasse, G.; Chambers, S.; et al. Acceptability and Preliminary Efficacy of a Web- and Telephone-Based Personalised Exercise Intervention for Individuals with Metastatic Prostate Cancer: The ExerciseGuide Pilot Randomised Controlled Trial. *Cancers* **2021**, *13*, 5925. https://doi.org/10.3390/cancers13235925.
22. Tabacof, L.; Delgado, A.; Dewil, S.; Reis, F.; Velar, C.M.; Corteline, M.E.D.; Cecatto, R.B.; Putrino, D.; Imamura, M.; Battistella, L.R.; et al. Safety and Feasibility of Outpatient Rehabilitation in Patients With Secondary Bone Cancer: A Preliminary Study. *Rehabil. Oncol.* **2021**, *39*, E42. https://doi.org/10.1097/01.REO.0000000000000241.
23. Zopf, E.M.; Newton, R.U.; Taaffe, D.R.; Spry, N.; Cormie, P.; Joseph, D.; Chambers, S.K.; Baumann, F.T.; Bloch, W.; Galvão, D.A. Associations between Aerobic Exercise Levels and Physical and Mental Health Outcomes in Men with Bone Metastatic Prostate Cancer: A Cross-Sectional Investigation. *Eur. J. Cancer Care* **2017**, *26*, e12575. https://doi.org/10.1111/ecc.12575.
24. Bourke, L.; Gilbert, S.; Hooper, R.; Steed, L.A.; Joshi, M.; Catto, J.W.F.; Saxton, J.M.; Rosario, D.J. Lifestyle Changes for Improving Disease-Specific Quality of Life in Sedentary Men on Long-Term Androgen-Deprivation Therapy for Advanced Prostate Cancer: A Randomised Controlled Trial. *Eur. Urol.* **2014**, *65*, 865–872.
25. Cheville, A.L.; Moynihan, T.; Herrin, J.; Loprinzi, C.; Kroenke, K. Effect of Collaborative Telerehabilitation on Functional Impairment and Pain Among Patients With Advanced-Stage Cancer: A Randomized Clinical Trial. *JAMA Oncol.* **2019**, *5*, 644–652. https://doi.org/10.1001/jamaoncol.2019.0011.
26. Dawson, J.K.; Dorff, T.B.; Todd Schroeder, E.; Lane, C.J.; Gross, M.E.; Dieli-Conwright, C.M. Impact of Resistance Training on Body Composition and Metabolic Syndrome Variables during Androgen Deprivation Therapy for Prostate Cancer: A Pilot Randomized Controlled Trial. *BMC Cancer* **2018**, *18*, 368. https://doi.org/10.1186/s12885-018-4306-9.
27. Scott, J.M.; Iyengar, N.M.; Nilsen, T.S.; Michalski, M.; Thomas, S.M.; Herndon, J., 2nd; Sasso, J.; Yu, A.; Chandarlapaty, S.; Dang, C.T.; et al. Feasibility, Safety, and Efficacy of Aerobic Training in Pretreated Patients with Metastatic Breast Cancer: A Randomized Controlled Trial. *Cancer* **2018**, *124*, 2552–2560. https://doi.org/10.1002/cncr.31368.
28. Solheim, T.S.; Laird, B.J.A.; Balstad, T.R.; Stene, G.B.; Bye, A.; Johns, N.; Pettersen, C.H.; Fallon, M.; Fayers, P.; Fearon, K.; et al. A Randomized Phase II Feasibility Trial of a Multimodal Intervention for the Management of Cachexia in Lung and Pancreatic Cancer. *J. Cachexia Sarcopenia Muscle* **2017**, *8*, 778–788. https://doi.org/10.1002/jcsm.12201.
29. Uster, A.; Ruehlin, M.; Mey, S.; Gisi, D.; Knols, R.; Imoberdorf, R.; Pless, M.; Ballmer, P.E. Effects of Nutrition and Physical Exercise Intervention in Palliative Cancer Patients: A Randomized Controlled Trial. *Clin. Nutr.* **2018**, *37*, 1202–1209. https://doi.org/10.1016/j.clnu.2017.05.027.
30. Uth, J.; Hornstrup, T.; Schmidt, J.F.; Christensen, J.F.; Frandsen, C.; Christensen, K.B.; Helge, E.W.; Brasso, K.; Rørth, M.; Midtgaard, J.; et al. Football Training Improves Lean Body Mass in Men with Prostate Cancer Undergoing Androgen Deprivation Therapy. *Scand. J. Med. Sci. Sports* **2014**, *24* (Suppl. 1), 105–112. https://doi.org/10.1111/sms.12260.
31. Villumsen, B.R.; Jorgensen, M.G.; Frystyk, J.; Hørdam, B.; Borre, M. Home-Based "exergaming" Was Safe and Significantly Improved 6-Min Walking Distance in Patients with Prostate Cancer: A Single-Blinded Randomised Controlled Trial. *BJU Int.* **2019**, *124*, 600–608. https://doi.org/10.1111/bju.14782.
32. Sprave, T.; Rosenberger, F.; Verma, V.; Förster, R.; Bruckner, T.; Schlampp, I.; Bostel, T.; Welzel, T.; Akbaba, S.; Rackwitz, T.; et al. Paravertebral Muscle Training in Patients with Unstable Spinal Metastases Receiving Palliative Radiotherapy: An Exploratory Randomized Feasibility Trial. *Cancers* **2019**, *11*, 1771. https://doi.org/10.3390/cancers11111771.
33. Rief, H.; Akbar, M.; Keller, M.; Omlor, G.; Welzel, T.; Bruckner, T.; Rieken, S.; Häfner, M.F.; Schlampp, I.; Gioules, A.; et al. Quality of Life and Fatigue of Patients with Spinal Bone Metastases under Combined Treatment with Resistance Training and Radiation Therapy- a Randomized Pilot Trial. *Radiat. Oncol.* **2014**, *9*, 151. https://doi.org/10.1186/1748-717X-9-151.
